# Mosquito-Borne Diseases Emergence/Resurgence and How to Effectively Control It Biologically

**DOI:** 10.3390/pathogens9040310

**Published:** 2020-04-23

**Authors:** Handi Dahmana, Oleg Mediannikov

**Affiliations:** 1 Aix Marseille Univ, IRD, AP-HM, MEPHI, 13005 Marseille, France; handy92@hotmail.fr; 2 IHU-Méditerranée Infection, 13005 Marseille, France

**Keywords:** mosquito-borne disease, pest control, insecticide resistance, biological control, paratransgenesis, *Wolbachia*, *Asaia*, *Bacillus*

## Abstract

Deadly pathogens and parasites are transmitted by vectors and the mosquito is considered the most threatening vector in public health, transmitting these pathogens to humans and animals. We are currently witnessing the emergence/resurgence in new regions/populations of the most important mosquito-borne diseases, such as arboviruses and malaria. This resurgence may be the consequence of numerous complex parameters, but the major cause remains the mismanagement of insecticide use and the emergence of resistance. Biological control programmes have rendered promising results but several highly effective techniques, such as genetic manipulation, remain insufficiently considered as a control mechanism. Currently, new strategies based on attractive toxic sugar baits and new agents, such as *Wolbachia* and *Asaia*, are being intensively studied for potential use as alternatives to chemicals. Research into new insecticides, Insect Growth Regulators, and repellent compounds is pressing, and the improvement of biological strategies may provide key solutions to prevent outbreaks, decrease the danger to at-risk populations, and mitigate resistance.

## 1. Introduction

The significant connection between fauna and flora in the world today is due to many factors, including the highest increase ever experienced in population growth accompanied by the evolution of transport systems. These factors disrupt biogeographic barriers and are followed by the first appearance of species in novel habitats [1,2]. In the Americas, incursions of these species are estimated to cause more than $120 billion in damage every year [3].

Deadly pathogens and parasites may be transmitted by arthropods [4], and the increasing global human and animal populations are threatened by such epidemics and pandemics [5]. Mosquitoes (Diptera: Culicidae) represent the most threatening vector due to their role in the transmission of dangerous pathogens [1]. Through trade and travel, key mosquito species are being introduced into novel habitats [2,6,7].

A number of chemical products formulated to provide a high safety profile are commercially available, but their toxicity to human skin and the nervous system can lead to several serious problems, such as rashes, swelling, and eye irritation [8]. The most important drawback of these products is the incidence of insecticide resistance, which has increased rapidly in recent years [9], and the extremely challenging or downright impossible task of finding and treating all mosquito breeding sites. New approaches and vector-control tools targeting aquatic stages and adults are urgently needed [10].

In this review, we discuss the current state of knowledge about mosquito-borne diseases and the latest figures from these resurgences, highlighting current techniques for their control and their limitations. We then focus on new innovative alternatives currently known but rarely used, others that are not used at all, and those that are still in the test or design phase but are very promising, which we suggest to be considered in the biological control of mosquito-borne diseases.

## 2. Resurgence of Diseases Transmitted by Mosquitoes

The three main mosquito genera, *Anopheles*, *Aedes*, and *Culex*, transmit the causative agents of numerous important diseases to humans as well as animals [11,12,13,14]. In this chapter, we briefly describe the resurgence of essential disease agents transmitted by mosquitoes and their impact on humans and animals.

**Figure 1:**
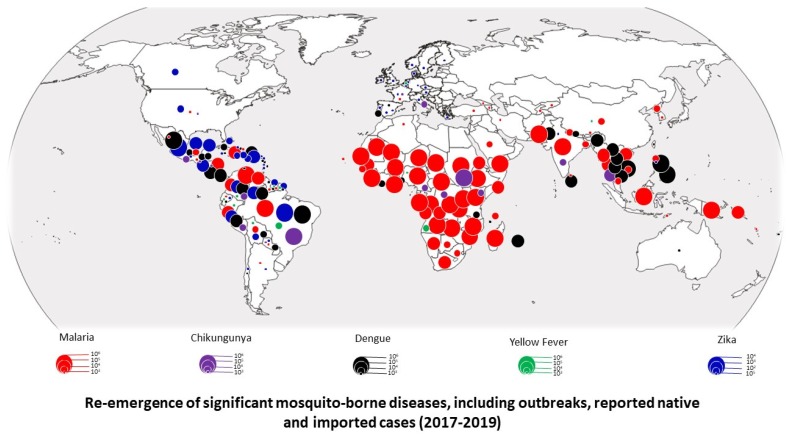
Cartography of significant resurgences of mosquito-borne diseases worldwide (until September 2019). We listed all reported outbreaks and imported and autochthon cases of malaria, dengue fever, yellow fever, chikungunya fever, and Zika fever between 2017 and 2019. This figure clearly shows their resurgence in almost all tropical countries. In many cases they were imported to several northern countries where the competent vector has become established, which may lead to potential local transmission.

Malaria is considered the most important parasitic disease of human beings and is currently endemic and transmitted by anopheline mosquitoes in more than 80 countries inhabited by approximately three billion people (Table S1; Figure 1), especially in sub-Saharan Africa, where more than 85% of cases and 90% of deaths occur, mainly in children younger than 5 years old. Malaria continues to cause phenomenal damage to public health (228 million cases worldwide, with 213 million (93%) reported in Africa alone, and severe outbreaks have recently ravaged many areas [15,16,17,18,19].

*Wuchereria bancrofti* and *Brugia* spp. can be transmitted by numerous mosquito species [13,20,21,22,23,24,25] (Table S1), and cause various clinical manifestations (25 million men with hydrocele and over 15 million people with lymphoedema) and at least 36 million people continue to have these chronic disease manifestations [26]. However, it is clear that eliminating lymphatic filariasis is not possible without controlling their vectors.

Dengue virus (DENV): Flaviviridae is responsible for dengue disease, caused by four distinct serotypes. Currently, it is the predominant arthropod-borne viral disease affecting humans [27], with 3.6 billion people living in areas at risk of transmission and hundreds of millions of dengue fever cases reported each year [28,29], causing ongoing epidemics in several countries [29,30] (https://www.outbreakobservatory.org/outbreak-thursday) (Table S1; Figure 1)

Zika virus (ZIKV): Flaviviridae also causes ongoing epidemics in several countries in Latin America and the Pacific [30,31,32,33,34] (https://www.who.int/emergencies/diseases/zika/en/) (Figure 1). *Aedes aegypti* is considered to be the primary vector associated with ZIKV outbreaks [35], while *Ae. albopictus* is considered a secondary vector [36]. However, several other species are also involved in the occurrence and transmission of this rapidly spreading virus [34,37,38] (Table S1). Currently, it is considered one of the most serious diseases threatening public health.

Chikungunya virus (CHIKV): Togaviridae is the causal agent of chikungunya fever (CHIKF) (Figure 1), known for producing an antalgic stance gait with severe articular pain [39]. Infected patients evolving to the chronic stage may range from 1.4% to 90% (52% in the American continent) [39]. Numerous outbreaks have recently been reported in several countries [30,40,41,42].

Yellow fever virus: Flaviviridae [43] is a haemorrhagic and potentially lethal RNA virus that causes outbreaks in several countries, especially in unvaccinated populations [44,45,46,47,48] (Table S1). Its emergence is cyclical, and outbreaks occur approximately 7–10 years apart [49]. In the summer of 2016, 47 countries declared YFV endemic, and 42 countries identified a risk of transmission, with 29 of them in Africa in 2017 [45]. With the highest fatality rate of up to 33.6%, numerous outbreaks continue to be registered [44,50]. Vaccination is safe, affordable, and the most effective way to prevent YF: “70 to 90 million doses are annually produced worldwide” [45].

Annually, the WHO reports approximately 67,000 cases of Japanese encephalitis, 20% to 30% of which are fatal, while 30% to 50% of survivors have significant neurological sequelae [51]. New strains genetically close to strains involved in previous outbreaks continue to be identified [52]. The St. Louis encephalitis virus was the major cause of epidemic encephalitis by an arbovirus in the USA [53]. It is re-emerging, causing numerous cases [54] (Table S1).

Similar to humans, horses are the domesticated animal that is most commonly affected by West Nile virus; 80% of cases are asymptomatic, while neurological signs are the most commonly reported symptom, with 90% of the 20% developing clinical signs, and the mortality rate may reach 30% [55]. Nevertheless, recent outbreaks in humans have been highlighted [56,57].

Different pathogenic blood-borne bacteria are regularly detected in mosquitoes [58,59]. It is not yet clear whether the presence of these bacteria in mosquitoes may be explained by occasional ingestion with blood meals or acquisition from the environment, or whether these bacteria may multiply and eventually be transmitted during blood meals. Different pathogenic alpha-proteobacteria, including *Anaplasma* spp., *Ehrlichia* spp., *Candidatus* Neoehrlichia, *Bartonella* spp., and *Rickettsia* spp., have been identified (xeno-monitoring studies) in adult mosquitoes [59,60]. More interestingly, the agent of febrile rickettsiosis, *Rickettsia felis*, has not only been identified in mosquitoes [58,61] but also shown to be potentially transmitted by *Anopheles* mosquitoes in laboratory experiments [62]. *Francisella tularensis* [63] is also carried by mosquitoes (*Aedes*), which act as a main vector in Sweden and Finland, making it the first reported mosquito-borne bacterium [63].

Several complex factors may explain the expansion of these diseases, such as population growth, globalisation of the economy, international travel (recreational, business, and military), inadequate vector-control efforts, limited access to good healthcare, rapid and unplanned urbanisation of tropical regions coupled with poor sanitary conditions, and a deterioration of public health infrastructures, all of which are related to climate change [64]; but, the major factors remain the mismanagement of insecticide use and the emergence of resistance.

## 3. Actual Insecticide-Based Vector-Control Strategies

The debate regarding dichlorodiphenyltrichloroethane (DDT) use for prevention, especially for malaria control, is polarised because it saved millions of lives worldwide but is unsafe. This has led to the invocation of precautions to enable choices to be made for healthier lives [65]. Some studies have focused on predicting mosquito abundance and assessing aquatic and adult mosquito control strategies [66], but despite the added efforts to develop new insecticides, other new alternative classes are slowly emerging [67,68].

### 3.1. Indoor Residual Spraying (IRS)

This is a well-developed and effective but potentially underused approach in vector control. It consists of treating the surfaces upon which common mosquitoes rest inside houses with a long-lasting insecticide. The most affected species among the endophilic species will be *Ae. aegypti*, which rests mainly indoors, feeds on humans, and is thus more likely to be reached by IRS than by space sprays [69]. IRS has some limitations and imperfections, such as the need for specialised training, which is time consuming in terms of obtaining public acceptance within a region. It does not prevent people from being bitten but above all, it must be adapted to several factors specific to a region, such as insecticide resistance, which is expensive and takes several years [70]. IRS has had a considerable impact on the mortality of *Ae. aegypti*, and used alone [71] or in combination with larval control [72] contributed to the elimination of *Ae. aegypti* in Guyana and the Cayman Islands, respectively [70]. In 2006, the WHO reaffirmed the importance of IRS for malaria transmission control, which was supported by the President’s Malaria Initiative (PMI) in 2012 [73]. Malaria eradication campaigns using IRS in the Mediterranean region seem to have led to the elimination of malaria [74]. New formulations could last between five and eight months [70]. The potential evolution of insecticide resistance in the vector to pyrethroids can be controlled using alternative formulations, such as bendiocarb [75] and other new IRS formulations [67,73,76,77]. Good insecticide management is based on an alternation of formulations to combat the evolution of resistance, which may maintain efficacy over time, especially for location-specific interventions [70].

### 3.2. Peridomestic Space Spraying

This strategy is attractive because it is highly visible and conveys the message that health authorities use vector-control activities [78]. The risks to humans due to the management of adult mosquitoes are probably negligible [79]. This has no direct impact on immature stages (egg, larvae, or pupae) [80], targeting adult mosquitoes only, and is performed by spraying small droplets of insecticide into the air. It is used mainly in emergency situations to limit the massive production of adult mosquitoes, thus decreasing the risk of existing outbreaks expanding [78].

To perform this intervention, two forms of space sprays are commonly used for control: thermal fog and cold fog, also known as ultralow volume (ULV) sprays. Both can be distributed using a vehicle-mounted or hand-held machine [78]. The insecticide concentration ranges from 2% (pyrethroids) to 95% (organophosphates), depending on the amount of active ingredient in the formulation. The applied volume is dependent on the compound concentration and toxicity to the target species [80]. Aerial spraying of pyrethrin significantly impacts small organisms found in the sprayed zones, which is not the case on large bodies [81].

For dengue control, mosquitoes emerging after treatment can still be vectors because the viruses can be transmitted transovarially. Therefore, their exposure to successive treatments seems necessary and should be done at intervals shorter than the extrinsic incubation period of the virus [82].

A high resurgence of mosquitoes was reported after six days of ULV treatment as a single method in Thailand [81], while good results were observed with a decrease in the incidence of dengue fever after a large emergency vector-control campaign included several space sprays [83].

### 3.3. Long-Lasting Insecticide-Treated Nets (LLINs)

Designed as a solution to the problems of conventional insecticide-treated nets (ITNs), and based on novel fabric technologies [84], LLINs were developed to resist multiple washes and remain effective for a prolonged time (at least three years). LLINs are considered one of the most successful mosquito control tools, especially for malaria prevention [85]. ITNs with synthetic pyrethroid insecticides either incorporated into or coated around their fibres have resulted in a considerable decline in malaria morbidity and mortality in several countries, especially in sub-Saharan Africa, where over 427 million nets were delivered between 2012 and 2014 [85]. Th annual cost of an LLIN can be as high as US$2.6, while IRS costs about US$4, and standard ITN costs range from US$1.5 to US$6 [85]. The level of use of LLINs varies according to several factors, such as temperature, humidity, season, and, especially, the density of mosquitoes, and access to them plays a major determinant of their use [86,87]. LLINs have contributed to the reduction in malaria over the past 15 years, combined with other new control measures, such as IRS and artemisinin-based combination therapies [88] in children and pregnant women [87,89]. Other important advantages of LLINS include reduced consumption of insecticides and insecticide released into the environment because they do not need retreatment [85]. The efficacy of LLINs is closely related to the molecules used (the choice depends on the presence or absence of its resistance) [90], and their correct use may enhance their efficiency [91]. In a study, the use of LLINs led to a dramatic reduction (97%) in the prevalence of malaria compared to a group of LLIN non-users [92].

### 3.4. Mosquito Repellents

Mosquitos are mostly attracted to humans by the lactic acid and CO_2_ present in our sweat that are detected by chemoreceptors present in their antennae, and repellents mask the human scent [8]. DEET (N,N-diethyl-meta-toluamide) is the most widely used and effective repellent against mosquitoes [93].

Biobased mosquito repellents are pest management tools that are based on safe, biologically based active ingredients derived from plants [94,95], fungi [96], or bacteria [93].

In terms of the effective control of mosquitoes and to ensure human and environmental safety where endemic mosquito resistance and environmental concerns limit the use of products, the use of biobased natural mosquito repellents is preferable to that of chemical repellents [8].

The most effective synthetic repellents are DEET (N,N-diethyl-m-toluamide) and IR3535 (3-(NButyl-N-acetyl)-aminopropionic acid [97]. Several nanoparticles synthesised and successfully impregnated into cotton fabrics in insect-repellent clothing show high efficacy against mosquito larvae and adult populations, which gives them the potential to be used as eco-friendly approaches to control mosquitoes if applied in long-lasting insect-repellent clothing [98,99]. The fact that the use of synthetic repellents causes insecticide resistance in mosquitoes, has a harmful effect on non-target organisms, and threatens the environment has led to widespread discussions around this method of control [97].

The increased involvement of governments and authorities on scientific projects coupled with correct individual action may help to combat the spread of mosquito-borne diseases and limit their devastating transmission.

## 4. Biological Control

Every year, promising new “eco-friendly” compounds are developed to progressively replace the oldest compounds, which are the most toxic and harmful. The use of biological control programmes, such as genetic modification or biological agents such as predatory fish, bacteria, protozoa, nematodes, and fungi, have rendered some promising results.

### 4.1. Genetic Modification

The sterile insect technique (SIT) is a species-specific and environmentally benign method for insect population control based on mass rearing, radiation-mediated sterilisation, and the release of a large number of male insects into a given target area, which compete for mates with wild males. A wild female mating with a released sterile male has no or fewer progeny, so the population tends to decline [100,101,102,103], which was an improvement on RIDL (release of insects carrying a dominant lethal gene). The lethal dominant gene could be controlled by a female-specific promoter and its expression could be inactivated by antibiotic treatment (tetracycline), allowing the mosquito-colony to be maintained. When male and female separation is required, the antibiotic is removed from the system, causing the death of all females [10]. Some projects cost approximately US$1.1 million [104], and some reports of failure have been published [105]. Mosquito egg production and mass rearing problems were also highlighted [106,107].

Paratransgenic strategies based on genetically modified symbiotic bacteria reintroduced in mosquitoes reveal a very high potential of casually controlling all-important mosquito species, including *Culex*, which is difficult to transform [14]. New studies on RNAi-based bioinsecticides (RNA interference) show promising results [108].

### 4.2. Fungi

Particular attention has been paid to fungal species belonging to the genera *Lagenidium*, *Coelomomyces*, *Entomophthora*, *Culicinomyces*, *Beauveria*, and *Metarhizium* for their power to reduce mosquito populations, but unfortunately, none of them have been specifically adapted as larvicidal agents against important vector species [109,110,111], even transgenic ones [112]. Application to surfaces on which mosquitoes land or need to pass through, such as fungus-impregnated cloths around bed nets, attractive bait stations, and adult mosquito traps and PET traps, show promising results, with a 39–50% reduction in survival rates of malaria-carrying mosquitoes and elimination of 95% of *Anopheles arabiensis* mosquitoes in a bait station [113]. One of the most effective fungi studied recently against simultaneously *Ae. albopictus* and *Cx. pipiens* mosquito adults is *Beauveria bassiana*. The production and persistence of its conidia was remarkably high [109]. 

### 4.3. Control of Aquatic Stages Using Elephant Mosquito and Fish Predators

The use of fish to control the aquatic stages of mosquitoes was an important tool in the pre-DDT era. These fish were introduced into all potential mosquito-breeding habitats and their use decreased after the introduction of DDT and then was rekindled after the development of resistance and harmful effects [114]. The use of indigenous larvivorous fishes is suggested [115], and a limited number of species are used, primarily *Gambusia affinnis* and *Poecilia reticulata*, although several failures have been reported in the literature [114]. Other aquatic predators may play a role in reducing mosquito populations, especially in rainy periods [116,117], and the combination of multiple predators can reduce mosquito populations [118].

The naturally occurring non-biting *Toxorhynchites* species, which exhibit predatory behaviour during their larval stages, have been explored for their potential use as biological control alternatives to chemical insecticides (the 4th instar larva is the most predaceous) [119,120]. Important progress was made concerning their production for use as biological agents and they demonstrated remarkable effectiveness against numerous mosquito species, such as *Ae. aegypti*, *Ae. albopictus*, and *Cx. quinquefasciatus* [119,121]. In certain situations, they have demonstrated practical potential, but their use continues to be limited by several problems, such as cannibalism during the early instars, temperature (limited by low temperatures), and also the inadequate overlap in the larval habitats between the prey and the predator mosquito [120].

### 4.4. Protozoan Control

*Chilodonella uncinata* is a protozoan parasite with many beneficial properties associated with a good microbial pathogen [122]. It causes low to very high (25–100%) mortality in mosquito larvae. It exhibits high virulence and resistance to desiccation and also demonstrates a high reproductive potential when cultured in vitro. Through its mosquito host, *C. uncinata* has the ability to spread in nature by the way of transovarian transmission [122].

## 5. Bacterial Agents Tested or Used in Control Strategies

Most of the attention of pest control scientists focuses on bacterial agents targeting both aquatic and adult stages. Several studies have shown their efficiency, and their use is recommended by the WHO. Here, we list some bacterial agents currently in use or undergoing tests with promising results. 

### 5.1. Bacillus spp.

**Figure 2:**
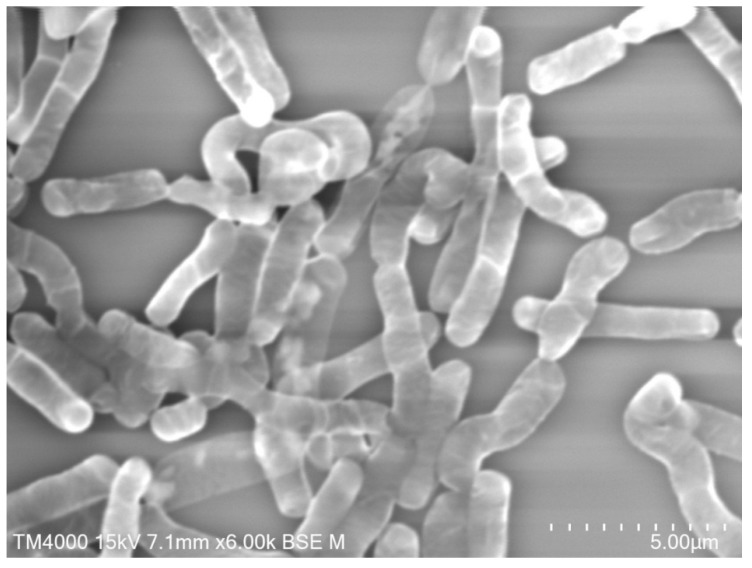
*Bacillus thuringiensis israelensis* (*Bti*), 3 days of culture, 4.9 µm in length (Hitachi TM4000) (personal image).

**Figure 3:**
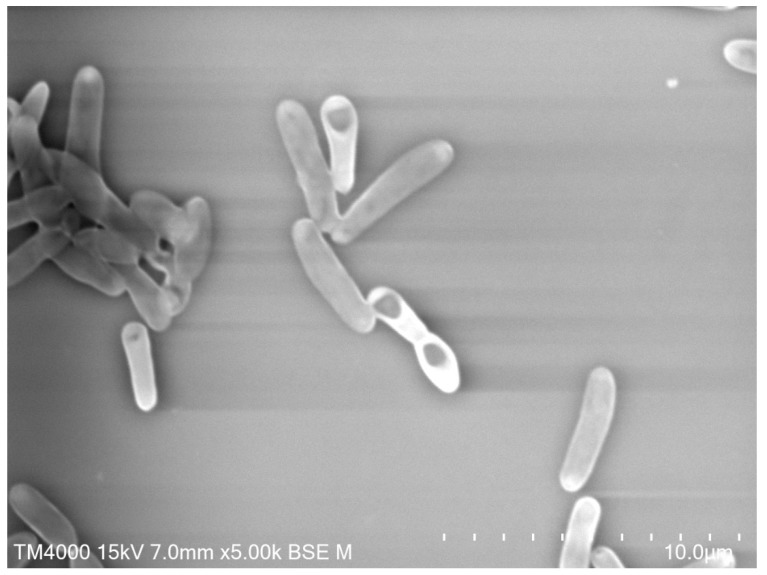
*Lysinibacillus sphaericus* (*Bs*) strain CSURP827, 3 days of culture, 5.02 µm in length (Hitachi TM4000) (personal image).

Before the discovery of *Bacillus thuringiensis israelensis* (*Bti*) (Figure 2) and *Bacillus sphaericus* (*Bs*) (Figure 3), little attention was paid to bacteria as sources of agents for microbial control of mosquitoes. Around 1500 microorganisms were recently identified as good potential insecticidal agents, and looking for insecticidal activity, metabolites from approximately a thousand microbial isolates were examined [123]. *Bti* formulations are the predominant nonchemical means employed for controlling mosquito larvae [124]. In addition, several studies indicate the highly effective and safe use of individuals or the mixture of *Bti* and *Bs* for mosquito control, and they are considered to be safe to non-target organisms cohabiting with mosquito larvae [125].

*B. thuringiensis* produces three classes of larvicidal proteins: *Cry* (exert intoxication through toxin activation, receptor binding, and pore formation in a suitable larval gut environment), *Cyt* (cytolytic toxicity) when sporulating (parasporal crystals), and *Vip* proteins throughout the vegetative phase (ionic, non-ionic detergents and pore-forming mechanisms of action were suggested), some of which are toxic against a wide range of insect orders, nematodes, and human-cancer cells. This has been widely employed as an effective biopesticide to control pests that are harmful to crops, forests, and humans. *Cyt* toxins possess less toxicity against mosquito larvae than *Cry* toxins [126,127].

Several species of *B. thuringiensis* exhibit a high mortality rate toward all mosquito larval instars, such as *Bti* [128], *B. thuringiensis* var. *krustaki* [129], *B. thuringiensis* var. *jegathesan* (*Btjeg*) [130], *B. thuringiensis* var. *kenyae*, and *B. thuringiensis* var. *entomocidus* [131]. Other species with homology to *Bacillus* show the highest toxicity against dipterans, such as *Clostridium bifermentans* (serovar *malaysia*) [132], *B. circulans* [133], and *B. laterosporus* [134,135]. *Bacillus* spp. remains a massive source of active compounds against pests, which are currently being explored to fill public health needs.

### 5.2. Insect Growth Regulators (IGRs)

Due to several advantages, such as low toxicity to the environment and selectivity, IGRs present an effective tool to control mosquito populations. They are substances that are analogues or antagonists of hormones and interfere with insect development [136]. There is growing interest in the use of IGRs, such as methoprene and pyriproxyfen, two juvenile hormone agonists belonging to IGR insecticides. They are effective against mosquito larvae and may inhibit the emergence of adults [137]; others include novaluron and diflubenzuron [138] for mosquito control [139]. Numerous recent studies have highlighted that mosquitoes and other pests have developed resistance to commonly used IGRs, such as methoprene and pyriproxyfen [140,141,142], which reinforces the need to develop new compounds and identify new targets in mosquitoes [143].

### 5.3. Wolbachia spp.

Mosquito symbiont-associated bacteria may exert a pathogenic effect on their host, interfering with its reproduction and also reducing vector competence [144]. *Wolbachia* are endosymbiotic bacteria that naturally infect approximately 40% of insect species [145,146], and *Wolbachia pipientis* is a unique valid species of the genus [147]. They are present in some major mosquito disease vectors, such as *Cx. quinquefasciatus*, *Ae. albopictus*, and anopheline species, including malaria vectors such as *An. gambiae* and *An. coluzzii* but never *Ae. aegypti* [144,145,148,149,150]. This maternally transmitted bacterium allowing the invasion of host populations can induce feminisation of males (turning genetic males into females), parthenogenesis (reproduction without males) [144], and cytoplasmic incompatibility, leading to the generation of inviable offspring when a *Wolbachia*-infected male mates with an uninfected female, but not in the contrary case [145]. Successfully used in Myanmar in the 1960s to eradicate *Cx. quinquefasciatus* [151], it is currently also being used to target *Ae. albopictus*, using a triple *Wolbachia*-infected strain [152], and to target *Ae. polynesiensis* (2012) [153]. To date, it has been used in several countries, such as Australia, Brazil, Indonesia, Vietnam, and Colombia. The fear of resistance to the inhibitory effect of *Wolbachia* has been highlighted, but no studies have demonstrated that this scenario is likely to happen, and the creation of *Wolbachia*-superinfected lines, such as *Ae. aegypti* with stable infection, could help to mitigate potential resistance [145,154] and add to their role in reducing vector competence. Studies have reported that *Wolbachia* inhibits the transmission of CHIKV [155], YFV [156], malaria parasites in *An. stephensi* [157] and *An. gambiae* [158], DENV [159], and ZIKV [160]. Recent reviews clearly explain *Wolbachia* as a form of biological control [144,161,162].

*Wolbachia*-based control constitutes a potentially promising strategy for the control of mosquitoes and their transmitted diseases that urgently needs to be considered and associated with biological control programmes in countries suffering from malaria and arbovirus outbreaks.

### 5.4. Asaia

To make malaria vectors inefficient, interruption of the cycle within the vector to stop parasite development before the *Anopheles* host becomes infective is a good solution [163]. The simplest approach to this is paratransgenesis, consisting of producing bacterial strains that are able to both live in the midgut of various mosquito species and spread rapidly among wild mosquito populations [164]. Several studies have been performed on the identification and use of competent microorganisms to combat vector-borne diseases [165]. The genus *Asaia*, first discovered in plant nectar, is an excellent candidate [166]; it is localized in many organs of mosquitoes, and can disperse inside the mosquito body through the haemolymph [165,167]. Its distribution in the mosquito population is made possible through several mechanisms (co-feeding, sexual mating, paternal, maternal, and horizontal transmission) [168,169,170]. *Asaia* bacteria may be genetically modified in order to be recolonised in a new host, resulting in spread within wild populations [166]. Recently, it was isolated and characterised from several *Anopheles* species, which would be beneficial if applied toward achieving paratransgenesis against malaria [165]. Advanced studies recently showed that *Asaia* may activate the mosquito’s immune system, leading to a reduction in the development of malaria parasites [171]. In the future, additional assets to which the bacterium may be used in mosquito control may be identified because it seems that *Asaia* plays a key role in the health of the mosquito host, even during its larval stage, allowing the larvae to develop rapidly [172]. Engineering of *Asaia* to produce an antiplasmodial effector causing the mosquito to become refractory to *Plasmodium berghei* is a perfect demonstration of the power of a transgenic microbiota [173], which makes it beneficial to microbial ecology and a potential candidate not only for paratransgenesis but also for general control of mosquitoes and mosquito-borne diseases.

### 5.5. Spinosyns

**Figure 4:**
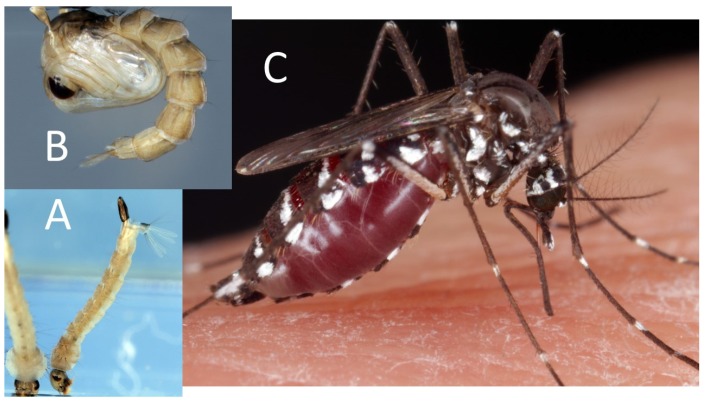
*Aedes albopictus* strain: (**A**) larvae (personal images), (**B**) pupa (personal images), and (**C**) adult (personal images).

**Figure 5:**
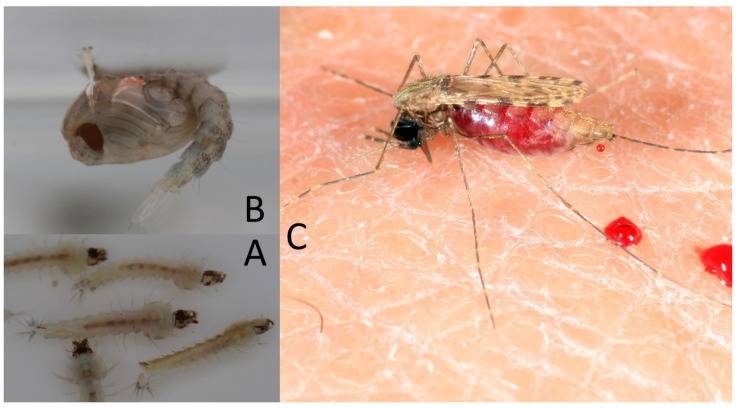
*Anopheles gambiae*: (**A**) larvae (personal images), (**B**) pupa (personal images), and (**C**) adult (personal images).

**Figure 6:**
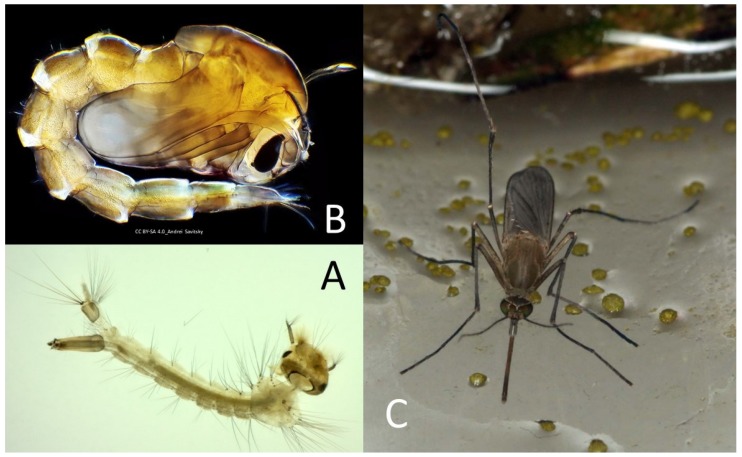
*Culex pipiens*: (**A**) larvae (personal images), (**B**) pupa (CC BY-SA 4.0_Andrei Savitsky), and (**C**) adult (personal images).

Spinosad is a biopesticide derived via fermentation from an actinomycete, *Saccharopolyspora spinosa*, a naturally occurring soil-dwelling bacterium. It contains two insecticidal factors, A (C_41_H_65_NO_10_) and D (C_42_H_67_NO_10_) [174,175]. It is categorised as a Group 5 insecticide by the Insecticide Resistance Action Committee (IRAC), forming a new class of polyketide-macrolide insecticides that act as nicotinic acetylcholine receptor (nAChR) allosteric modulators. Discovered in the 1980s in an early-stage insecticide screen that included *Ae. aegypti*, it was shown to be highly active against numerous pests in the Lepidoptera, Diptera, Thysanoptera, Coleoptera, Orthoptera, and Hymenoptera orders, and others. Its application to mosquito control is relatively new due to its pesticidal activity after ingestion and cuticle absorption and its highly favourable toxicology profiles in mammals and the environment [176]. It was also recently approved for use as a mosquito larvicide in human drinking water sources and containers [177]. Its applications in natural habitats are too few, but in laboratories it has been demonstrated to be very efficient at preventing and reducing larval development in important medical and veterinary vector species, such as *Ae. aegypti*, *Ae. albopictus* (Figure 4), *Anopheles gambiae* (Figure 5), *An. pseudopunctipennis*, *An. albimanus*, *Cx. pipiens* (Figure 6), *Cx. quinquefasicatus* [175,178], and some anopheline species [179,180].

As the best solution, biological control requires several components for the design of effective plans for mosquito control, and spinosad, which has no resistance because it was introduced recently into control programmes, will play a very important role.

### 5.6. Bacterial-Based Feeding Deterrents and Repellents

The fear of the occurrence of possible side effects of DEET, such as toxic encephalopathy, seizures, acute manic psychosis, cardiovascular toxicity, and dermatitis [181], as well as potential resistance that has become a reality with *Ae. aegypti* mosquitoes [182] and *An. gambiae* [183], has led to the use of innovative technologies to create other products free of DEET that are marketed in the form of sprays or creams and include other active ingredients [184], such as picaridin [185] and IR 3535 [186], and a wide range of essential oils, which synergistically use various components and have been reported to provide a higher repellent activity than single isolated components [187]. Recently, a mixture of compounds isolated from *Xenorhabdus budapestensis* (entomopathogenic-associated bacteria) exhibited potent feeding-deterrent activity against three mosquito species considered to be the most important vectors of diseases affecting public health. They belong to the fabclavine class and exhibit a high activity comparable to or better than that of DEET or picaridin in side-by-side assays [93], which supports the attempt to replace toxic molecules by considering bacteria as a very promising source of new alternative molecules for exploitation as mosquito repellents.

## 6. Biological Insecticide Resistance

In view of their efficacy and safety, the importance of bacterio-insecticides seems to be increasing in insect control activities, which has led researchers to investigate and characterise new bacterial strains with insecticidal properties and identify their active compounds.

### 6.1. Resistance to Bti

Numerous factors, such as wild proliferation or environmental accumulation, as well as the persistence of human-spread *Bti* in treated larvae breeding sites, may lead to a long exposure time of insects, which may increase the risk of acquiring resistance in target insects and also have a negative impact on non-target insects [188]. A study showed that a high resistance to each individual *Bti* toxin can be obtained under some conditions in the laboratory after only a few generations of selection, and this resistance seems to be lowest for commercial and environmental *Bti*, which might act as a first step in resistance to a complete *Bti* toxin mixture (Table 1). Studies reporting that individuals show resistance to one toxin but not to another suggest that different resistance mechanisms exist [189]. The mechanisms of resistance to *Bti Cry* toxins are widely studied in *Culex* and *Aedes* species [176,190,191,192,193,194], and marginal cross-resistances have been identified [193,195]. To date, resistance of malaria-carrying species to *Bti* has not been found [196].

### 6.2. Resistance to Bs

*B. sphaericus* (*Bs*) is found in numerous habitats, especially in soils and aquatic habitats. It is known as a producer of a characteristic spherical spore inside the swollen sporangium. Over the past 25 years, scientists had much interest and focused on isolating numerous strains because of their potential use as mosquito larvicides [200]. Several formulations used in biocontrol are highly effective against mosquitoes [125]. Recently renamed *Lysinibacillus sphaericus* (2007) [201], numerous studies have reported various levels of resistance to *Bs* in laboratory and field populations from different countries [176] (Table 1), mostly on *Culex* populations. Different mechanisms [202] have been observed in numerous locations, such as France, China, India, and Brazil [126,203]. If mosquitoes develop resistance to one strain of *Bs*, it appears that they will develop resistance to other *Bs* strains due to the similarity of the binary toxins in most strains; but, they remain susceptible to *Bti* [176]. Resistant strain fitness was found to be heavily impacted, especially fecundity and fertility, which became very low in a study [204,205], although opposite results were achieved in another study [206].

Although it has been tested widely for controlling malaria vectors [207,208,209,210], no laboratory or field resistance has been highlighted for *Anopheles* species to date.

### 6.3. Resistance Management

When it comes to managing the rapid increase in insecticide resistance [9], *Bti* can be used as a powerful tool to mitigate resistance to *Bs* in mosquitoes, although it has been reported that using them in rotation or in a mixture leads to a steady decline in resistance over 30 generations. They can also delay or prevent the emergence of resistance due to the synergistic action between their toxins, and recent formulations have shown greater larvicidal activity and efficacy [176]. Other combinations with botanical pesticides are considered alternatives to mitigating the development of resistance to *Bs* in mosquitoes [211].

The best solution for the management of insecticide resistance is to systematically replace most of the molecules used in chemical control with eco-friendly biological control, but that strategy will depend on the plans conceived, the number of molecules chosen and the associations between the various formulations to prevent and reduce current resistance and avoid the appearance of new resistance.

## 7. Current Challenges for a Prosperous Future

In view of the current situation and the failures that have been experienced with mosquitos invading new territories associated with devastating outbreaks, new tools, molecules, plans, synergistic associations, and methods of mosquito control are being developed to facilitate strategic objectives, such as protecting at-risk populations, especially in endemic areas; preventing the international spread of mosquitoes and the diseases they carry; and rapidly containing epidemics. Some strategies are in the stage of preliminary testing or in the validation phase and others have recently been introduced into use.

### 7.1. New Insecticide, IGR, and Repellent Compounds

The most urgent need is to develop new insecticides to fight mosquito-borne diseases due to their crucial efficiency and their economic importance [212]. The Innovative Vector Control Consortium (IVCC) has released new product classes, especially for malaria eradication, and manages international efforts to establish new methods, including producing a new ATSB (attractive targeted sugar bait) product class and programming next-generation IRS projects [213]. Due to their eco-friendly properties and efficiency, entomopathogenic Ascomycete fungi have been suggested for the control of both larval and adult stages of dengue vectors [12,110]. Several other bacteria showing promising results on numerous pests have been suggested to have the same effect on mosquitoes, such as the entomopathogenic nematode-associated bacteria *Xenorhabdus* sp. [93,214]; *Serratia marcescens*, which is often associated with insect infection and shows high insecticidal effects alone [215] or when associated with other insecticides [216]; and entomopathogens [217]. Other bacteria exhibiting toxic effects on mosquitoes, such as *Clostridium bifermentans* [132], may also be considered in control strategies.

Recently, a new compound class, chalcones with JHAN activity, showed impressive insecticide and IGR activity when tested against *Ae. albopictus* larvae and could be useful for the development of environmentally benign IGR insecticides to control mosquitoes [143]. Moreover, the beneficial effects of diterpene and their derivatives as well as their potential use as biological alternatives in dengue fever control has been highlighted [218].

Auto-dissemination is a phenomenon where the dispersal and transfer of active compounds is carried out by contaminated adult mosquitoes to treat undeveloped habitats that are difficult to locate and treat [219]. It can occur through treated materials or dissemination stations, such as modified ovitraps, and can also be combined with other methods, such as SIT [12], which may increase their effectiveness.

### 7.2. Attractive Toxic Sugar Baits (ATSB)

Bait aims to attract mosquitoes in order to feed them on toxic sugar meals broadly sprayed on plants or placed in bait stations [220,221]. They show the highest efficacy in laboratory and field studies [222] against *Aedes* species, culicines, and sand flies [12]. 

Whether for indoor or outdoor control, ATBS can reduce mosquito populations through direct mortality caused by feeding them on insecticide-treated bait but also through the spread of mosquito pathogens or non-chemical toxins [223]. Developing mosquito-specific attractants to avoid their effects on non-target species make baits one of the best solutions, and their combination with other strategies, such as genetic ones, will maximise their effectiveness.

### 7.3. Parasitic Nematodes

Lot of nematodes belonging to numerous orders and families are known to be parasites of insects [224]. Some insect parasitic nematodes that are specific to mosquitoes [225] may be considered alternatives to chemical insecticides [226]. When tested, they were effective against malaria vectors and several other important mosquito species, such as *Ae. aegypti*, *Ae. albopictus*, *Cx. quinquefasciatus*, and *An. gambiae* [226,227,228,229]. As they are naturally adapted to their host, such nematodes are highly specific to theirs hosts, which they can kill by producing high levels of parasitism. They are free swimming and disseminate easily in the infective stage [225], and species such as *Romanomermis iyengari* are widely suggested to be a component of integrated mosquito control programmes in lymphatic filariasis endemic countries [229].

### 7.4. Acoustic Larvicides and Traps

These are emerging technologies designed to combat the aquatic stages of mosquitoes by killing them with sound waves resulting in instantaneous mortality or inhibited emergence. They have proven to be effective as a beneficial non-chemical alternative for the treatment of drinking water supplies [230]. This approach has been shown to be highly effective in a range of typical volumes found in peri-domestic water containers [230] without causing resistance within mosquito populations or harming non-target organisms when used properly [231]. Furthermore, even simple and cheap mobile phones can sensitively acquire acoustic data on the species-specific level of adult wingbeat sounds. This makes it possible to simultaneously record the time and location of the encounter between humans and mosquitos, which forms a powerful tool for acoustically mapping mosquito species distribution worldwide [232]. Other innovative acoustic-based tools have been developed to control mosquitoes during rear-and-release operations, such as the low-cost and battery-powered sound-baited gravid *Aedes* trap, which may be an effective replacement for the costly Biogents Sentinel (BGS) trap [233].

### 7.5. Advanced Genetic Studies

Recently, a new RNAi-based bioinsecticide was developed from D-RNA molecules, which was subsequently tested on *Aedes* larval breeding water [108]. A significant reduction in the viability of the larvae treated with dsRNA was reported while in the surviving larvae and adults, altered morphology and chitin content was observed. In combination with diflubenzuron, this innovative bioinsecticide had insecticidal adjuvant properties [108]. 

In another study, a considerable reduction in the fertility of *Ae. aegypti* adult males was observed when feeding their larvae double-stranded RNAs (dsRNAs) targeting testis genes. Moreover, several dsRNAs were reported to be inducing males and were remarkably effective in competing for mates. RNAi-mediated knockdown of the female-specific isoform of double-sex was also effective in producing a highly male-biased population of mosquitoes, making it possible to overcome the need to sex-sort insects before release [234].

## 8. Conclusions

Despite currently deployed methods, epidemics and the spread of mosquito-borne diseases continue as a result of a range of complex reasons, including insecticide resistance, inappropriate design of control programmes, ineffective coverage, missing and poorly trained manpower, as well as a lack of financial resources and infrastructure [12].

Many strategies have been designed for the control of mosquito-borne diseases, each with their strengths and weaknesses. However, approaches such as integrated vector management that adopts receding horizon control strategies, which may consider multiple objectives, seem to provide optimal control solutions that are fast and sustainable but that also offer the most cost-effective control choices [235]. 

Improving current strategies, such as the sterile insect technique, the release of insects with dominant lethality, or transgenesis, may provide key solutions to preventing outbreaks, decreasing the danger to at-risk populations and mitigating resistance. Meanwhile, promising techniques, such as those discussed in this manuscript, have already proven their effectiveness but remain under-used and require more attention and consideration in vector-control plans. 

## Figures and Tables

**Table 1:** Highlights of field and laboratory insecticide resistances to *Bti* and *Bs*.

Bacteria	Mosquito	Site	Type of Study	Number of Studied Regions	Date	Reference
*Bti* + *Bs*	*Culex pipiens*–complex	Onondaga County, USA	Field	2	June 2003	[140]
*Bti*	*Ochlerotatuscataphylla*	Rhône-Alpes, France	Field	4	April 2003	[197]
*Bti*	*Aedes rusticus*	Rhône-Alpes, France	Field	13	Winters 2005 and 2006	[198]
*Bti*	*Culex quinquefasciatus*	USA	Laboratory	1	Summer 1990	[191]
*Bti*	*Aedes aegypti*	USA	Laboratory	1	2011	[192]
*Bs*	*Culex pipiens*–complex	Utah, USA	Field	3	September 2016	[199]
*Bti*	*Aedes aegypti*	France	Laboratory	1	2010	[189]

## Supplementary Materials

The following are available online at https://www.mdpi.com/2076-0817/9/4/310/s1, Table S1: Updates concerning important mosquito borne diseases. We listed most of the mosquito-borne diseases, including their actual distribution, transmission, natural occurrence or animal infection, and virulence as well as the existence or absence of treatments or vaccines to date.
