# Supplementary material for: Mosquito-Borne Diseases Emergence/Resurgence and How to Effectively Control It Biologically
Source: Pathogens. 2020 Apr 23;9(4):310. doi: 10.3390/pathogens9040310 (PMC7238209; doi:10.3390/pathogens9040310)
Supplement: Supplementary file 1 [file pathogens-09-00310-s001.pdf]

Pathogens

Review

Mosquito-borne disease resurgence and how to effectively control it biologically

Handi Dahmana[1], Oleg Mediannikov[1]

Table S1: Updates concerning important mosquito borne diseases. We listed most of the mosquito-borne diseases, including their actual distribution, transmission, natural occurrence or animal infection, and virulence as well as the existence or absence of treatments or vaccines to date.

Pathogen	Disease	First isolation	Nature of pathogen	Distribution	Transmission	Other natural occurrence	Other infected animals	Virulence and symptoms	Vaccine and treatment	Economic impact
***West Nile Virus* (*WNV*)**	West Nile fever and encephalitis	1937 from a febrile patient in Uganda	Single-stranded RNA virus (*Flaviviridae*)	Endemic and widespread across tropical parts of Africa, southern Asia, and northern Australia and episodic in more temperate parts of Europe [1]	Mosquitoes: *Culex* spp (Main vector), *Aedes albopictus*, *Mansonia* spp, *Coquillettidia* spp, *Cx. univittatus*	Several bird species	Horses	Virulence differences between strains are genotype-specific and not associated with lineage (16). Infection develops between 3 and 14 days after the bite of a WNV-infected mosquito, and can persist for a further 3 to 6 days or up to 60 days in severe cases. When they occur, Symptoms can include fever, rash, headaches, muscle weakness and joint pains or hepatitis and 1% can develop signs of meningo-encephalitis including severe headache, flaccid paralysis and occasionally death. A prolonged recovery period of up to 60 days for patients seeking medical attention [2]	Not yet for people -Available for horses [3]	In 2005, the total economic impact of an outbreak of WNV in one state in USA was $2.98 million [4]
***La Crosse virus* (*LACV*)**	La Crosse encephalitis	1964, brain tissue of a 4-year-old girl, in La Crosse, Wisconsin, USA	Arbovirus (*Bunyaviridae*)	North America	*Ochlerotatus triseriatus*, *Aedes hendersoni*, *Ae. canadensis*, *Ae. communis*, *Ae. informatus*, *Culex pipiens*, *Cx. restuans* and *Orthopodomyia signifera*,	*	Chipmunks, deer, rabbits, red foxes, woodchucks, squirrels, raccoons, horses, cows, pigs and dogs [6]	The most pathogenic of the California encephalitis serogroup viruses, Clinical expression of the disease varies from 5 to 15 days after exposure to the virus, inducing symptoms including fever,	Not yet	3 million dollars per patient [7][8]

					Aedes triseriatus, *Aedes albopictus* and *Aedes japonica* [5]			headache, myalgia, and occasionally discomfort and prostration. In severe cases there is an encephalitis that causes significant neurological sequelae for life or in rare cases death (< 1% of cases) [6]		
Eastern Equine Encephalitis Virus (EEEV)	Encephalitis	In 1933, from an infected horse brain.--- The first description of the disease in humans was in 1972	Alphavirus (*Togaviridae*)	The Americas	*Culiseta melanura* and mosquitoes within the genera *Aedes*, *Culex*, and *Coquillettidia* -- Organ transplant [9]	*	Swine, cattle, white tailed deer, alpacas, emus, and pheasants, birds, snakes, canids and horses [10]	Mortality rates in humans and horses of 50–75% and 70–90% respectively, it become the most lethal of the naturally occurring encephalitic alphaviruses of North America, complications and residual neurologic deficits [11]	Vaccines are licensed for veterinary applications , no licensed vaccines for use in humans [12]	Treatment and long-term care for just a single case can reach several million dollars [9]
Western Equine Encephalitis Virus (WEEV)	Encephalitis	Described in 1930, the first case described in humans of the WEE were in 1941 in Canada and USA	Alphavirus (*Togaviridae*)	The Americas	*Culex tarsalis*, *Culiseta* and *Aedes* mosquito species, cross the placenta and blood transfusion transmission [13]	*	Birds are a reservoir serving as amplifying hosts, horses, pheasants mules [13]	Acute inflammation of the brain parenchyma and meninges -- older adults mostly develop more severe neuro-invasive disease or to die then infants and very young children that when to them, they have 30% of chance to develop a permanent disability such as seizures, spasticity, and cognitive or behavioural disorders. Mortality is low, around 4% mostly fatal in elderly patients. 3 to 50% of mortality in horses [13]	Vaccines are licensed for veterinary applications , no licensed vaccines for use in humans [12]	Treatment and long-term care for just a single case can reach several million dollars [9]
Venezuella equine encephalitis virus (VEEV)	Encephalitis	In 1938 in a horse in Venezuela, the disease was described in Venezuela in the 1920s and in Colombia in 1967 [12]	*Alphavirus* (*Togaviridae*)	The Americas	Isolated from *Aedes*, *Culex, Psorophora* spp and *Ae. taeniorhynchus* [12]	*	Rodents- horses [12]	VEE complex alphaviruses are classified into six subtypes, designated I to VI, and consist of 9 species -- cause severe febrile illness which may develop to encephalitis evolving to cause severe human morbidity and mortality. Permanent neurologic sequelae - Mortality in humans (1%) but it can reach 80% in horses [12]	Vaccines are licensed for veterinary applications , no licensed vaccines for use in humans [12].	Treatment and long-term care for just a single case can reach several million dollars [9]
Jamestown Canyon Virus (JCV)	Encephalitis	In 1961 from *Culiseta inornata* mosquitoes in Jamestown Canyon, Colorado, USA	Enveloped single-stranded RNA virus with tripartite genome.	The temperate north America	*Jamestown Canyon virus* was isolated from 22 mosquito species [14]	*	*	The causal agent of moderate to severe central nervous system disease, especially for adults ones. Fever, Generalized weakness,	Not yet	*

								headache, myalgia, nausea, neck rigidity, altered mental status and seizures mostly reported from neuroinvasive and non neuroinvasives cases [15]		
St. Louis Encephalitis virus (SLEV)	St. Louis Encephalitis	In 1933 in St. Louis, Missouri, USA	(*Flaviviridae*)	Throughout the Americas and the Caribbean	Organ transplant, *Culex* mosquitoes, the genera A*edes*, *Coquillettidia*, *Deinocerites*, *Mansonia*, *Psorophora*, S*abethes* and *Wyeomyia* [16]	*	Columbiform birds as amplificatory hosts, Small birds (family *Formicariidae*), lake cingulates, bats, folivores, rodents and marsupials [17]	Neuroinvasive disease, range from non-specific febrile syndrome to febrile headache, aseptic meningitis and encephalitis with fatality ranges from 3-30% [18]	Not yet	*
Chikungunya virus (CHIKV)	Chikungunya	In 1952 in Tanzania	Enveloped, positive, single-stranded RNA virus that belongs to the *Togaviridae* family, *Alphavirus* genus	Tropical and sub-tropical areas	*Ae. albopictus* and *Ae. aegypti* mosquitoes. Transmission by maternal infections during gestation	*	*	The manifestations of the disease were divided in three clinical stages: The acute stage, from the first day until the 21th day of clinical symptoms, the post-acute stage, from the 21st day until the end of the third month of disease and the chronic stage, after 3 months of persisting articular complains. CHIKF infected patients evolving to the chronic stage may range from 1.4% to 90%, 52% in the American continent. The disease inflict an arthralgia which is often bilateral, symmetric and affects multiple, mostly distal joints, a high fever, a myalgia and headache, in addition to a skin rash, gastrointestinal symptoms, fatigue, asthenia, peripheral oedema and conjunctivitis [19]	No effective antiviral treatment, only symptomatic treatment exist.	Disability adjusted life years reached US$185 billion [20]
Dengue Virus (DENV)	Dengue disease	In 1944 in japan.	A single-strand positive-sense genome (approximately 10,700 bases). (*Flaviviridae*)	Tropical and sub-tropical areas.	*Ae. aegypti* (Linnaeus) mosquito, *Ae. albopictus*	*	*	Four distinct serotypes (DENV-1, -2, -3 or -4) -- Includes asymptomatic infection, mild dengue fever (DF), dengue haemorrhagic fever (DHF) and dengue shock syndrome, myocardiopathy, hepatic failure and neurological disorders. It's ranked actually the most prevalent	Dengvaxia [23]	Around $2.1 billion per year [24]

								arboviral disease of humans [21][22]		
***Zika virus* (*ZIKV*)**	Zika disease. Can cause microcephaly.	In 1947 in a sentinel rhesus monkey in Uganda -- first reported human illness case caused by the ZIKV was in 1954 during an outbreak of jaundice in Nigeria	Genus *Flavivirus* 53 virus. (*Flaviviridae*)	The Americas, Africa, southern Asia and some countries in Europe [25]	Thirty-one wild-caught mosquito species infected with *ZIKV* worldwide [25]. During pregnancy, sexual contact, breastfeeding, or blood transfusion [26]	*	*	During the infection, we can find hematospermia, hearing difficulties, thrombocytopenia and subcutaneous bleeding and Guillain-Barré syndrome which is a neuromuscular complication, meningoencephalitis and acute myelitis but the major complication is the microcephaly with surrounding 8301 cases in less than one year (2015-2016) in Brazil [27]	Vaccines are currently in clinical trials [28]. Only symptomatic treatment [29].	U$3.5 billion in 2016 [30]
***Yellow fever virus* (*YFV*)**	Yellow fever	First reports occurred since 500 years ago, 1927 in Ghana	Haemorrhagic and a potentially lethal RNA virus. (*Flaviviridae*)	Tropical and subtropical areas of sub-Saharan Africa and Central and South America (47 countries declared YFV as endemic)	*Haemagogus* spp. and *Sabethes* spp. (sylvatic cycle) and *Aedes aegypti* (urban cycle)	*	Monkeys	Symptoms appears 3 to 6 days after infected mosquito bite, characterized by 3 stages, the infection period, when patients can present malaise, headaches, photophobia, lumbosacral pain, pain in the lower extremities, myalgia, anorexia, nausea, vomiting, restlessness, irritability, and dizziness, than the remission period which lasts about 48 hours and many patients recover after this stage, only about 15% will progress until the third stage which is the intoxication period characterized by returning signs and symptoms including fever, prostration, nausea, vomiting, epigastric pain, jaundice, oliguria, haemorrhagic diathesis and possibly organ failure (80). with high fatality rate (30 to 50%) (29 000 to 60 000 deaths annually) [31]	No specific antiviral therapy available. Vaccination, is safe, affordable, and the most effective way to prevent YF (70 to 90 million doses are annually produced worldwide)	US$18.5 billion per year [32]
Plasmodium falciparum* or *Plasmodium vivax*, but also by *Plasmodium ovale	Malaria	Malaria has been in existence for 50,000–100,000 years (Charles Louis	Parasite	Endemic in 108 countries inhabited by roughly 3 billion people especially in sub-	*Anopheles* mosquitoes which is the most important (*Anopheles gambiae* complex), transfusion of infected blood	*	Monkeys and rodents	After Malarial infected mosquito bite, the incubation period can range from 9 to 40 days during which the patient is asymptomatic,	Malaria is a preventable and treatable disease, Artemisinin combination given for 3 days [35].	US$8 billion per year [37]

curtisi*, *Plasmodium ovale wallikeri*, *Plasmodium malariae and Plasmodium knowlesi		Alphonse Laveran, 1880) Nobel prize for physiology medicine		Saharan Africa w	or the use of contaminated needles and the Transplacental passage [33]			its shortest for *P. falciparum* while its longest for *P. malariae* and patients present fatigue, headache, arthralgia, myalgia, abdominal and chest pain which are not specific to Malaria disease, then followed by fever which is often high, spiking up to 40 C° in children and non-immune individuals and in *P. vivax* infection can be associated with intense rigors they may also present nausea, vomiting, diarrhoea, backpain, pallor and jaundice and in 50% of cases in children an acute gastroenteritis, upper respiratory tract infection and rarely a jaundice mimicking viral hepatitis. Splenomegaly more commonly, hepatomegaly and pallor are distinctive physical findings and no specific symptoms differentiating Malaria infection from other diseases --85% of the cases and 90% of deaths occurs, mainly in children younger than 5 years (abortion or infant mortality)--in 2015 alone, there were an estimated 214 million new cases of malaria worldwide and 438,000 deaths [34]	Malaria vaccine development is in progress [36]	
Wuchereria bancrofti, ***Brugia malayi***, ***B. timori***, ***B. pahangi***, ***Dirofilaria repens*** and ***Dirofilaria immitis***	Filariasis	*	Parasite	Tropical and subtropical areas of Asia, Africa, the Western Pacific and some parts of the Americas	*Culex*, *Anopheles*, *Aedes* and *Mansonia* genera. *Cx. pipiens pallens*, *Cx. pipiens molestus*, *Cx. pipiens quinquefasciatus*, *Cx. vagans*, *Cx. whitmorei* and *Oc. togoi*, *Cx. bitaeniorhynchus*, *Cx. Pseudovishnui*. *Cx. sinensis*, *Cx. tritaeniorhynchus*, *An. sinensis* and	*	Carnivors especially dogs	Acute fevers, chronic lymphedema, elephantiasis and hydrocele and which results in the loss of 5.9 million adjusted life by year- Psychological impacts --120 million humans affected . 40 million of chronically disabled people across 55 countries are estimated [38]	Mass Drug Administration (MDA) (preventive chemotherapy , MDA for at least five years), Triple-drug therapy using Diethylcarbamazine (DEC), Albendazole (ALB) and Ivermectin [39]	*

					Armigeres subalbatus					
Francisella tularensis	Tularemia	In 1912 by McCoy and Chapin as Bacterium tularense	Gram-negative coccobacillus, an aerobic bacterium. It is nonspore-forming, nonmotile [40,41]	Sweden and Finland [42]	*Aedes* spp. [42]	*	Found in more 200 species of different animals--several arthropods (ticks)	*	No FDA approved vaccine. Development in progress [43]. Ciprofloxacin and gentamycin 7 to 14 days [44].	*
Japanese encephalitis virus (JEV)	Japanese encephalitis (JE)	*	A single-strand positive-sense genome (*Flaviviridae*)	Present in Asia, from Japan to India and Pakistan [45]	*Culex* spp.	*	Ardeid birds, bats and pigs	67,000 cases of JE, 20 % to 30 % of them are fatal while 30% to 50% of survivors have significant neurological sequelae [46]	Preventable disease by vaccines [47]. Treatment is symptomatic	The numbers-needed-to-treat to prevent a case and cost per case averted were approximately 0.7 million and $0.6 billion for Risk Category I, 1.6 million and $1.2 billion for Risk Category II, and 9.8 million and $7.6 billion for Risk Category III [48].

*: Not provided = not studied, not reported or not found

References:

1. Kilpatrick, A.M. Globalization, land use and the invasion of West Nile virus NIH Public Access. *Science (80-.).* **2011**, *334*, 323–327.

2. Sule, W.F.; Oluwayelu, D.O.; Hernández-Triana, L.M.; Fooks, A.R.; Venter, M.; Johnson, N. Epidemiology and ecology of West Nile virus in sub-Saharan Africa. *Parasites and Vectors* **2018**, *11.*

3. West Nile virus Available online: https://www.who.int/en/news-room/fact-sheets/detail/west-nile-virus (accessed on Mar 30, **2020**).

4. Barber, L.M.; Schleier, J.J.; Peterson, R.K.D.; Peterson, R.K.D. Economic cost analysis of West Nile virus outbreak, Sacramento County, California, USA, 2005. *Emerg.*

Infect. Dis. **2010**, *16*, 480–6.

5. Camille Harris, M.; Yang, F.; Jackson, D.M.; Dotseth, E.J.; Paulson, S.L.; Hawley, D.M. La crosse virus field detection and vector competence of culex mosquitoes. *Am. J. Trop. Med. Hyg.* **2015**, *93*, 461–467.

6. Harding, S.; Greig, J.; Mascarenhas, M.; Young, I.; Waddell, L.A. La Crosse virus: A scoping review of the global evidence. *Epidemiol. Infect.* **2019**, *147*.

7. S. Harding, J. Greig, M. Mascarenhas, I.Y. and L.A.W. La Crosse virus: a scoping review of the global evidence. *Epidemiol. Infect.* **2018**.

8. Barber, L.M.; Schleier, J.J.; Peterson, R.K.D. Economic cost analysis of West Nile virus outbreak, Sacramento County, California, USA, 2005. *Emerg. Infect. Dis.* **2010**, *16*, 480–486.

9. Armstrong, P.M.; Andreadis, T.G. Eastern equine encephalitis virus - Old enemy, new threat. *N. Engl. J. Med.* **2013**, *368*, 1670–1673.

10. Hubálek, Z.; Rudolf, I.; Nowotny, N. Arboviruses Pathogenic for Domestic and Wild Animals. *Adv. Virus Res.* **2014**, 201–275.

11. Ronca, S.E.; Dineley, K.T.; Paessler, S. Neurological sequelae resulting from encephalitic alphavirus infection. *Front. Microbiol.* **2016**, *7*.

12. Forrester, N.L.; Wertheim, J.O.; Dugan, V.G.; Auguste, A.J.; Lin, D.; Adams, A.P.; Chen, R.; Gorchakov, R.; Leal, G.; Estrada-Franco, J.G.; et al. Evolution and spread of Venezuelan equine encephalitis complex alphavirus in the Americas. *PLoS Negl. Trop. Dis.* **2017**, *11*.

13. Simon, L. V.; Gossman, W.G. *Encephalitis, Western Equine*; **2018**;

14. Andreadis, T.G.; Anderson, J.F.; Armstrong, P.M.; Main, A.J. Isolations of Jamestown Canyon virus (Bunyaviridae: Orthobunyavirus) from field-collected mosquitoes (Diptera: Culicidae) in Connecticut, USA: A ten-year analysis, 1997-2006. *Vector-Borne Zoonotic Dis.* **2008**, *8*, 175–188.

15. Vosoughi, R.; Walkty, A.; Drebot, M.A.; Kadkhoda, K. Jamestown Canyon virus meningoencephalitis mimicking migraine with aura in a resident of Manitoba. *CMAJ* **2018**, *190*, E262–E264.

16. Beranek, M.D.; Gallardo, R.; Almirón, W.R.; Contigiani, M.S. First detection of *Mansonia titillans* (Diptera: Culicidae) infected with St. Louis encephalitis virus (Flaviviridae: Flavivirus) and Bunyamwera serogroup (Peribunyaviridae: Orthobunyavirus) in Argentina. *J. Vector Ecol.* **2018**, *43*, 340–343.

17. Kopp, A.; Gillespie, T.R.; Hobelsberger, D.; Estrada, A.; Harper, J.M.; Miller, R.A.; Eckerle, I.; Müller, M.A.; Podsiadlowski, L.; Leendertz, F.H.; et al. Provenance and geographic spread of St. Louis encephalitis virus. *MBio* **2013**, *4*.

18. Curren, E.J.; Lindsey, N.P.; Fischer, M.; Hills, S.L. St. Louis encephalitis virus disease in the United States, 2003-2017. *Am. J. Trop. Med. Hyg.* **2018**, *99*, 1074–1079.

19. Couzigou, B.; Criquet-Hayot, A.; Javelle, E.; Tignac, S.; Mota, E.; Rigaud, F.; Alain, A.; Troisgros, O.; Francois, S.P.; Abel, S.; et al. Occurrence of chronic stage chikungunya in the general population of martinique during the first 2014 epidemic: A prospective epidemiological study. *Am. J. Trop. Med. Hyg.* **2018**, *99*, 182–190.

20. Tiina M. Murtola; S. S. Vasan; Tapasvi I. Puwar; Dipti Govil; Robert Field; Hong-Fei Gong; Ami Bhavsar; Jose Suaya; Marion Howard; Donald S. Shepard; et al. Preliminary estimate of immediate cost of chikungunya and dengue to Gujarat, India.

Dengue Bull. 34 32-39 **2010**, 32–39.

21. Ramos-Castañeda, J.; Barreto Dos Santos, F.; Martínez-Vega, R.; Lio, J.; Galvão De Araujo, M.; Joint, G.; Sarti, E. Dengue in Latin America: Systematic Review of Molecular Epidemiological Trends. **2017**.

22. Kulkarni, A. V.; Choudhury, A.K.; Premkumar, M.; Jain, P.; Gupta, E.; Sarin, S.K. Spectrum, Manifestations and Outcomes of Dengue Infection in Individuals with and without Liver Disease. *J. Clin. Transl. Hepatol.* **2019**, *7*, 1–6.

23. Flasche, S.; Jit, M.; Rodríguez-Barraquer, I.; Coudeville, L.; Recker, M.; Koelle, K.; Milne, G.; Hladish, T.J.; Alex Perkins, T.; T Cummings, D.A.; et al. The Long-Term Safety, Public Health Impact, and Cost-Effectiveness of Routine Vaccination with a Recombinant, Live-Attenuated Dengue Vaccine (Dengvaxia): A Model Comparison Study. *Plos Med.* **2016**.

24. Shepard, D.S.; Coudeville, L.; Halasa, Y.A.; Zambrano, B.; Dayan, G.H. Economic impact of dengue illness in the Americas. *Am. J. Trop. Med. Hyg.* **2011**, *84*, 200–207.

25. Gutiérrez-Bugallo, G.; Piedra, L.A.; Rodriguez, M.; Bisset, J.A.; Lourenço-de-Oliveira, R.; Weaver, S.C.; Vasilakis, N.; Vega-Rúa, A. Vector-borne transmission and evolution of Zika virus. *Nat. Ecol. Evol.* **2019**, *3*, 561–569.

26. Runge-Ranzinger, S.; Morrison, A.C.; Manrique-Saide, P.; Horstick, O. Zika transmission patterns: a meta-review. *Trop. Med. Int. Heal.* **2019**, *24*, 523–529.

27. Song, B.H.; Yun, S.I.; Woolley, M.; Lee, Y.M. Zika virus: History, epidemiology, transmission, and clinical presentation. *J. Neuroimmunol.* **2017**, *308*, 50–64.

28. WHO WHO | Zika virus vaccine product development. *WHO* **2019**.

29. CDC Treatment | Zika virus | CDC Available online: https://www.cdc.gov/zika/symptoms/treatment.html (accessed on Mar 30, **2020**).

30. Qureshi, A.I. Economic Impact of Zika Virus. In *Zika Virus disease*; Elsevier, 2018; pp. 137–142.

31. WHO Yellow Fever | WHO | Regional Office for Africa Available online: https://www.afro.who.int/health-topics/yellow-fever (accessed on Mar 30, 2020).

32. Ozawa, S.; Clark, S.; Portnoy, A.; Grewal, S.; Stack, M.L.; Sinha, A.; Mirelman, A.; Franklin, H.; Friberg, I.K.; Tam, Y.; et al. Estimated economic impact of vaccinations in 73 low-and middle-income countries, 2001-2020. *Bull World Heal. Organ* **2017**.

33. Blümel, J.; Burger, R.; Drosten, C.; Gröner, A.; Gürtler, L.; Heiden, M.; Jansen, B.; Klamm, H.; Ludwig, W.D.; Montag-Lessing, T.; et al. Malaria. *Transfus. Med. Hemotherapy* 2009, *36*, 48–60.

34. WHO Severe Malaria . *Trop. Med. Int. Heal.* **2014**.

35. WHO | Overview of malaria treatment. *WHO* **2020**.

36. Quadiri, A.; Kalia, I.; Kashif, M.; Singh, A.P. Identification and characterization of protective CD8 $^{+}$ T-epitopes in a malaria vaccine candidate SLTRiP. *Immunity, Inflamm. Dis.* **2020**, *8*, 50–61.

37. World Health Organzation *Global technical strategy for malaria 2016-2030. World Health Organisation*; 2015;

38. WHO Lymphatic filariasis Available online: https://www.who.int/news-room/fact-sheets/detail/lymphatic-filariasis (accessed on Mar 30, 2020).

39. Gyapong, J.O.; Owusu, I.O.; da-Costa Vroom, F.B.; Mensah, E.O.; Gyapong, M.

Elimination of lymphatic filariasis: current perspectives on mass drug administration. *Res. Rep. Trop. Med.* **2018**, *Volume 9*, 25–33.

40. *Francisella tularensis Colony Morphology*;

41. *Tularemia (Francisella tularensis)*;

42. Lundström, J.O.; Andersson, A.-C.; Bäckman, S.; Schäfer, M.L.; Forsman, M.; Thelaus, J. Transstadial transmission of Francisella tularensis holarctica in mosquitoes, Sweden. *Emerg. Infect. Dis.* **2011**, *17*, 794–9.

43. Bitsaktsis, C.; McCauley, J.; Devenney, K. Protective role of TH17 cells in an FcγR targeting immunization strategy against the respiratory bacterial pathogen, Francisella tularensis. *J. Immunol.* **2019**, *202*.

44. Bahuaud, O.; Le Brun, C.; Chalopin, T.; Lacasse, M.; Le Marec, J.; Pantaleon, C.; Nicolas, C.; Barbier, L.; Bernard, L.; Lemaignen, A. Severe infections due to Francisella tularensis ssp. holarctica in solid organ transplant recipient: Report of two cases and review of literature. *BMC Infect. Dis.* **2019**, *19*, 238.

45. Geographic Distribution of Japanese Encephalitis Virus | Japanese Encephalitis | CDC Available online: https://www.cdc.gov/japaneseencephalitis/maps/index.html (accessed on Mar 30, 2020).

46. Yin, Z.; Wang, X.; Li, L.; Li, H.; Zhang, X.; Li, J.; Ning, G.; Li, F.; Liang, X.; Gao, L.; et al. Neurological sequelae of hospitalized Japanese encephalitis cases in Gansu province, China. *Am. J. Trop. Med. Hyg.* **2015**, *92*, 1125–1129.

47. Taucher, C.; Kollaritsch, H.; Dubischar, K.L. Persistence of the immune response after vaccination with the Japanese encephalitis vaccine, IXIARO® in healthy adults: A five year follow-up study. *Vaccine* **2019**, *37*, 2529–2531.

48. Carias, C.; Hills, S.L.; Kahn, E.B.; Adhikari, B.B.; Fischer, M.; Meltzer, M.I. Comparative economic analysis of strategies for Japanese encephalitis vaccination of U.S. travelers. *Vaccine* **2020**, *38*, 3351–3357.
